# Validation of the Finnish version of the SCOFF questionnaire among young adults aged 20 to 35 years

**DOI:** 10.1186/1471-244X-9-5

**Published:** 2009-02-08

**Authors:** Sini Lähteenmäki, Terhi Aalto-Setälä, Jaana T Suokas, Suoma E Saarni, Jonna Perälä, Samuli I Saarni, Hillevi Aro, Jouko Lönnqvist, Jaana M Suvisaari

**Affiliations:** 1 Department of Mental Health and Substance Abuse Services, National Institute for Health and Welfare, Helsinki, Finland; 2 Helsinki University Central Hospital, Hospital for Children and Adolescents, Department of Adolescent Psychiatry, Helsinki, Finland; 3 Helsinki University Central Hospital, Department of Psychiatry, Helsinki, Finland; 4 Department of Public Health, University of Helsinki, Helsinki, Finland; 5 Department of Psychiatry, University of Helsinki, Helsinki, Finland; 6 Department of Social Psychiatry, Tampere School of Public Health, University of Tampere, Tampere, Finland

## Abstract

**Background:**

We tested the validity of the SCOFF, a five-question screening instrument for eating disorders, in a general population sample.

**Methods:**

A random sample of 1863 Finnish young adults was approached with a questionnaire that contained several screens for mental health interview, including the SCOFF. The questionnaire was returned by 1316 persons. All screen positives and a random sample of screen negatives were invited to SCID interview. Altogether 541 subjects participated in the SCID interview and had filled in the SCOFF questionnaire. We investigated the validity of the SCOFF in detecting current eating disorders by calculating sensitivity, specificity, and positive and negative predictive values (PPV and NPV) for different cut-off scores. We also performed a ROC analysis based on these 541 persons, of whom nine had current eating disorder.

**Results:**

The threshold of two positive answers presented the best ability to detect eating disorders, with a sensitivity of 77.8%, a specificity of 87.6%, a PPV of 9.7%, and a NPV of 99.6%. None of the subjects with current eating disorder scored zero points in the SCOFF.

**Conclusion:**

Due to its low PPV, there are limitations in using the SCOFF as a screening instrument in unselected population samples. However, it might be used for ruling out the possibility of eating disorders.

## Background

Eating disorders (ED) are a group of psychiatric disorders characterized by disordered eating habits and excessive focus on one's weight. They are most common in young females, among whom they present an important cause of physical and psychosocial morbidity. It has been estimated that 0.3–2.2% of young Western females suffer from anorexia nervosa [1-4] and 1–2% from bulimia nervosa [1,2,4]. Mortality from anorexia nervosa is as high as 5.0% [5]. Early detection and treatment improve the prognosis of eating disorders [6,7].

Detecting eating disorders early is difficult because of the cryptic presentation of the disorder. A simple screening method for detecting eating disorders would be very useful for primary care and for student healthcare. Such screen would be valuable for research purposes as well. A few screening tools have been developed [8-10], but they are often lengthy and may be difficult to interpret for a non-specialist. Recently, a new screening tool, the SCOFF questionnaire, was developed to overcome these limitations [6]. The SCOFF is a simple and memorable instrument of five questions intended to raise suspicion of an existing eating disorder. So far, only a limited number of studies concerning the validity of the SCOFF have been performed (table 1). The previous estimates of the sensitivity, specificity, positive predictive value, and negative predictive value of the SCOFF have varied between 53.3–100%, 21–94.4%, 24.4–81%, and 88.7–99.3%, respectively, depending on sample characteristics (table 1). While sensitivity and specificity are properties of the instrument, positive predictive value depends on both the test and the population in which it is used. Lower disease prevalence in the population leads to lower positive predictive value. Accordingly, positive predictive value has been best in studies of treatment-seeking young adults and in adolescents and lowest in studies on unselected primary care patients. The SCOFF has also been used in other recent studies which did not specifically focus on its validity as an eating disorder screen [11-14].

**Table 1:** Previous articles concerning the validity of the SCOFF:

Article	Population	Number of cases	Diagnostic procedure	Sensitivity (Threshold ≥2) %	Specificity %	PPV %	NPV %
The SCOFF questionnaire: assessment of a new screening tool for EDs Morgan, J 1999	Cases: Women confirmed as having anorexia or bulimia nervosa Controls: University students confirmed as not having an ED	116 96 total 214	SCOFF vs. clinical diagnosis based on DSM-IV* criteria	100	87.5	-	-

The SCOFF questionnaire and clinical interview for EDs in general practice: comparative study Luck, A 2002	Women attending two general practices in London	341	SCOFF vs. clinical diagnosis based on DSM-IV criteria	84.6	89.6	24.4	99.3

Validation of SCOFF questionnaire among pre-teenagers Caamaño, F 2002	One class of pre-teenagers selected randomly from 10 Spanish schools	289	SCOFF vs. EAT**	64.1	87.2	43.9	94.0

Four simple questions can help screen for EDs Cotton, M-A 2003	College students and patients from primary care clinics in London	129 104 total 233	SCOFF and ESP*** vs. Q-EDD**** (based on DSM-IV criteria)	78	88	-	-

Application of the SCOFF, EAT and TFEQ questionnaires in women seeking diet-therapy Siervo, M 2005	Women seeking diet-therapy at the outpatient dietetic clinic in Naples Remark: Only patients with BED, bulimic EDNOS or normal eating behaviour were selected in the study.	162	SCOFF, EAT and TFEQ***** vs. clinical diagnosis based on DSM-IV criteria	94	21	-	-

Eating Disorders in Graduate Students: Exploring the SCOFF as a simple screening tool Parker, S 2005	Graduate students attending the university student health clinic	297	SCOFF vs. EDE-Q ******	53.3	93.2	66.7	88.7

Validation of the SCOFF questionnaire for screening the eating behaviour disorders of adolescents in school Rueda, G 2005	Randomly selected students from three schools in Colombia	241	SCOFF vs. CIDI******* (based on DSM-IV criteria)	81.9	78.7	62.1	91.1

Validation of the SCOFF for screening of EDs in university women Rueda, G 2005	University students in Colombia	385	SCOFF vs. CIDI (based on DSM-IV criteria)	78.4	75.8	46.5	92.9

Validation of the Spanish version of the SCOFF questionnaire for the screening of EDs in primary care Garcia-Campayo, J 2005	Patient with probable eating disorder diagnosis from six primary health care centers in Spain.	203	SCOFF vs. SCAN******** (based on DSM-IV criteria)	97.7	94.4	81	93.1

Screening for EDs in primary care: EDE-Q versus SCOFF Mond, J 2008	Women aged 18–40 years attending two primary care practices in smaller urban regions of the USA	147	SCOFF and EDE-Q vs. telephone interview including diagnostic items of the EDE*********	72	73	35	-

*Diagnostic and Statistical Manual of Mental Disorders, fourth edition

**Eating Attitude Test

***Eating disorder Screen for Primary care

****Questionnaire for Eating Disorder Diagnoses

*****Three Factor Eating Questionnaire

******Eating Disorder Examination-Questionnaire

*******Composite International Diagnostic Interview

********Schedules for Clinical Assessment in Neuropsychiatry

********* Eating Disorder Examination interview

A general population study setting is appropriate for investigating whether a screen is suitable for detecting cases when there is no selection based on symptoms in the study population. In this study, we tested the validity of the SCOFF in screening current eating disorders in Finnish young adults. To our knowledge, this is the first study reporting the validity of the SCOFF in a population-based sample of young adults.

## Methods

### Participants

The Mental Health in Early Adulthood in Finland (MEAF) study (described also by Castaneda et al. [15] and Suvisaari et al[16]) is based on the Health 2000 Study [17-19], a nationally representative two-stage cluster sample of 1894 persons aged 18–29 years (the young adult sample) and 8028 persons aged 30 years and over (the adult sample) from 80 municipalities or groups of municipalities with joint primary care in Finland, including the 15 biggest towns. The Health 2000 sampling was done by Statistics Finland, and the sample size was determined so that it allowed investigation of the prevalences of most health problems by sex and age groups [17]. The baseline assessment of the young adult sample was conducted in 2001, and consisted of an interview and a questionnaire.

MEAF was a follow-up study of the Health 2000 young adult study sample. While the Health 2000 study covered all aspects of health, MEAF focused on mental health. A two-phase study design was used in the MEAF study. 2–4 years after the original study, a questionnaire was mailed to all members of the young adult sample excluding those who had died or refused further contacts. It included several scales assessing mental health and substance use. Persons reporting symptoms above a defined threshold in any screening scale were asked to participate in the mental health interview. These screens were: the General Health Questionnaire (GHQ-12) [20], the K10 [21], the CAGE [22], the CIDI section on psychotic symptoms [23,24], the Mood Disorder Questionnaire (MDQ) [25], the SCOFF [6], treatment contact for mental health problems, and history of suicide attempt. The SCOFF was used to screen current eating disorders, with a cut-off point of two or more positive answers. The SCOFF scale was translated into Finnish by the research team, and back-translated by an experienced medical language editor who is a native English speaker.

In addition, all Health 2000 young adults who had had hospital treatment because of any mental disorder (ICD-10 section F, ICD-8 and ICD-9 290–319) according to the Finnish Hospital Discharge Register information were asked to participate in the interview, along with a random subsample of Health 2000 young adults who were screen negative. Altogether, the screening questionnaire was sent to 1863 persons and returned by 1316 (70.6%). We invited 982 persons to the mental health assessment, and 546 (55.5%) completed the study protocol [16]. Of these 982 persons approached, 821 were screen positive in at least one of the mental health screens, and 161 were screen negative. The participants were not informed whether they were selected because of having psychological symptoms or whether they were screen negative controls.

The ethics committees of the National Public Health Institute and the Hospital District of Helsinki and Uusimaa approved the Health 2000 survey and the MEAF reassessment. Participants provided written informed consent according to the Declaration of Helsinki [17,18].

### Mental health assessment

The mental health assessment included structured questions on sociodemographic variables and treatment received for mental health and substance use disorders, a semi-structured psychiatric interview (SCID-I, research version) for diagnosing current (1-month) and lifetime mental disorders [26], neuropsychological assessment, and collection of blood sample for genetic analysis. The face-to-face interview was conducted by experienced research nurses and psychologists and all interviews were reviewed by a clinical supervisor. Participants who had had treatment contacts for mental health problems were asked for a permission to assess case notes from such contacts. Final diagnostic assessments according to DSM-IV-TR criteria were made by experienced clinicians (Jaana Suvisaari, Samuli Saarni, Jonna Perälä, and Terhi Aalto-Setälä) based on the interview and case note data.

In this validation study, the study sample comprised 541 subjects who had been interviewed and who had also filled in the SCOFF questionnaire. The eating disorders assessed were anorexia nervosa (AN), bulimia nervosa (BN), and eating disorder not otherwise specified (EDNOS). The EDNOS diagnosis was used for clinically significant disorders of eating that did not meet the criteria for anorexia or bulimia nervosa. Clinical significance was assessed by the interviewer together with a psychiatrist and confirmed in final diagnostic assessment using both interview and case note data. Examples included cases that met all except one criteria for anorexia or bulimia nervosa, and cases with binge eating disorder. The SCID-I interview produced both current and lifetime diagnoses of these disorders. We investigated the validity of SCOFF in detecting any current (present within the past month) eating disorder.

### The SCOFF screen

The SCOFF questionnaire contains five questions concerning eating habits and attitudes toward one's weight and body shape.

The acronym **SCOFF **is created from the questions: 1. Do you make yourself **S**ick because you feel uncomfortably full? 2. Do you worry you have lost **C**ontrol over how much you eat? 3. Have you recently lost more than **O**ne stone (6 kg in the Finnish version) in a 3 month period? 4. Do you believe yourself to be **F**at when others say you are too thin? 5. Would you say that **F**ood dominates your life? A threshold of 2 positive answers has been proposed to raise a suspicion of an existing eating disorder [6,27].

Of the subjects who had filled in the SCOFF questionnaire (n = 1303), 541 (312 women) participated in the mental health interview. Of them, 72 (13.3%) had scored at least two points and had thus been selected for the interview based on their SCOFF answers. There were 54 SCOFF screen positives (7.1%) among those who had not participated in the interview. There was no significant difference in the mean SCOFF score between those who had been interviewed and those who had been invited to the interview but refused to participate (0.48 vs. 0.58, t = 1.59, p = 0.11). When analyzing the SCOFF questions at the item level, the only significant difference between those who participated versus those who did not participate in the interview related to the item involving appearance: those who believed they were fat although others said they were too thin were less likely to participate. The frequency of this symptom among participants was 9.1% versus 13.4% in nonparticipants (χ^2 ^= 4.28, d.f. = 1, p = 0.039).

### Statistical analysis

Sensitivity is the proportion of persons with diagnosis who are detected positive by the test. Specificity is the proportion of persons without diagnosis who are detected negative by the test. Positive predictive value refers to the proportion of positive test results that are true positive, and negative predictive value refers to the proportion of negative test results that are true negatives. The Receiver Operating Characteristics (ROC) curve is a plot of the balance between sensitivity and specificity for a diagnostic test [28]. The closer the curve follows the left-hand border and then the top border of the ROC space, the more accurate the test, whereas the closer the curve comes to the 45-degree diagonal of the ROC space, the less accurate the test. The area under the curve is a measure of test accuracy.

The validity of the SCOFF was assessed by calculating the sensitivity, specificity, and positive and negative predictive values for screening cut-off points ≥1, ≥2, ≥3 and ≥4 positive answers. The calculations were carried out with Statistical Analysis Systems (SAS, Version 9.1) [29]. The ROC analysis was conducted using a web-based calculator designed by John Eng [30].

## Results

### Current eating disorders

In our sample, current eating disorders were detected in ten participants, nine of whom had filled in the SCOFF questionnaire. Table 2 summarizes sensitivity, specificity, positive predictive value, and negative predictive value of the SCOFF in detecting current eating disorders for different cut-off points. Setting the threshold at two or more positive answers to all five questions provided 77.8% sensitivity with specificity of 87.8%. A positive predictive value (PPV) of 9.7% was attained with a negative predictive value (NPV) of 99.6%.

**Table 2:** The validity of the SCOFF in detecting current eating disorders (men and women combined)

Threshold (number of positive answers)	Sensitivity % (95% Cl)	Specificity % (95% Cl)	Pos. Predictive value % (95% Cl)	Neg. Predictive value % (95% Cl)
≥ 1	100.0 (66.4–100.0)	70.9 (66.8–74.7)	5.5 (2.5–10.2)	100.0 (99.0–100.0)
≥ 2	77.8 (40.0–97.2)	87.8 (84.7–90.4)	9.7 (4.0–19.0)	99.6 (98.5–100.0)
≥ 3	33.3 (7.5–70.1)	97.4 (95.6–98.6)	17.7 (3.8–43.4)	98.9 (97.5–99.6)
≥ 4	22.2 (2.8–60.0)	99.6 (98.7–100.0)	50.0 (6.8–93.2)	98.7 (97.3–99.5)

Only one man was diagnosed with a current eating disorder. Therefore, we re-ran the analyses among women only. Among women, using two or more positive answers as the cut-off point produced sensitivity of 75.0%, specificity of 84.2%, PPV of 11.1%, and a NPV of 99.2% (table 3).

**Table 3:** The validity of the SCOFF in detecting current eating disorders (women only)

Threshold (number of positive answers)	Sensitivity % (95% Cl)	Specificity % (95% Cl)	Pos. Predictive value % (95% Cl)	Neg. Predictive value % (95% Cl)
≥ 1	100.0 (63–100.0)	64.1 (58.5–69.5)	6.8 (3.0–13.0)	100.0 (98.1–100.0)
≥ 2	75.0 (34.9–6.87)	84.2 (79.6–88.1)	11.1 (4.2–22.6)	99.2 (97.2–99.9)
≥ 3	25.0 (3.2–65.1)	95.6 (92.8–97.7)	13.3 (1.7–40.5)	98.0 (95.7–99.3)

**Figure 1:**
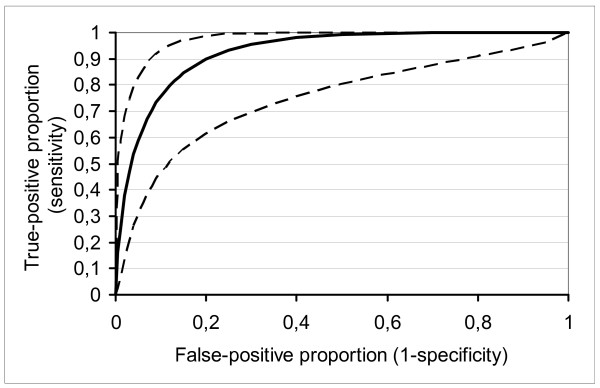
**The Receiver Operating Characteristic (ROC) curve (men and women combined)**. Fitted ROC Area: 0.926. Empiric ROC Area: 0.919. The solid line represents the ROC curve of the SCOFF, and the dashed lines represent 95% confidence intervals.

The Receiver Operating Characteristic (ROC) curve set the optimal threshold for the questionnaire at two or more positive answers (figure 1). With this cut-off, the fitted ROC area was 0.926 and the empiric ROC area 0.919.

## Discussion

SCOFF was designed to be a simple, memorable screening instrument for primary care, intended to raise suspicion that an eating disorder might exist before rigorous clinical assessment [6]. This is the first study in which the SCOFF is being evaluated in young adults from the general population. We found fairly similar sensitivity and specificity for the recommended SCOFF cut-off point as in previous studies conducted in primary care [27,31-33] or among students [31,32,34-36]. However, the same cut-off produced unacceptably low positive predictive (PPV) value for screening eating disorders from the general population. On the other hand, none of the subjects with current eating disorder scored zero point in the SCOFF, and thus SCOFF might be useful for ruling out eating disorder.

Our results concerning the sensitivity and specificity of the SCOFF when using two or more positive answers as the cut-off point were quite consistent with previous studies made with similar research frame [34-36] or with samples representing primary care patients [27,31-33]. However, PPVs were considerably better in these studies than in our study. The PPV is important especially when identifying relatively rare disorders such as eating disorders, because their detection can be ineffective among general population even with instruments with relatively high specificity and sensitivity [37]. The problem of low PPV has also been discussed previously by Mond and colleagues [33].

The SCOFF seems to work considerably better among risk population than among general population. The studies testing the SCOFF among patients with probable eating disorder diagnosis versus healthy controls [6,7,38] report almost exclusively excellent validity figures. It might be that these excellent figures are at least partly due to the research frame; the subjects are in easily recognizable advanced stages of the disease while the controls consist of healthy people only. This leads to a marked difference between these two groups and can therefore give overoptimistic results [37]. Even people attending general practice already differ from the general population because they present some symptoms and seek treatment for some problem. People with eating disorders consult general practitioners more frequently than healthy young adults even prior to the diagnosis [39] and thus there might be an over-representation of eating disorders in these study samples. In unselected population samples of young adults eating disorder prevalences are rather low; in our sample the prevalence estimates for lifetime anorexia nervosa and bulimia nervosa among women were 2.14% and 2.25%, respectively [40], which are comparable with previous estimates in studies with similar research frame [4].

It also might be that the SCOFF works best among adolescents. This would explain the greater PPV of the study made by Caamaño et al [34], in which the sample consisted of 11–13-year-olds.

Other screens for eating disorders in general (Stirling Eating Disorder Scales (SEDS), BEDT, Eating Disorder Diagnostic Scale (EDDS)) have shown PPVs comparable to our results, but population-based knowledge of these instruments is limited [41-44]. Mond et al reported relatively high PPV (30%) for Eating Disorder Examination Questionnaire (EDE-Q) in a population-based study of adult women [45]. Many screens are limited to a specific disorder like Eating Attitudes Test (EAT-40 and EAT-26) to anorexia nervosa and The Bulimic Investigatory Test (BITE) and (BULIT) to bulimia nervosa. Compared to SCOFF most of the screens mentioned above are longer and more difficult to interpret and have shown relatively low positive predictive values [46]. Our results are in line with Jacobi et al. suggesting that further research is needed to identify target populations for SCOFF [46]. The negative predictive value of the SCOFF has been excellent in this and previous studies, suggesting that it could be used to rule out eating disorder, while persons with at least some symptoms of disordered eating should be assessed in more detail.

There are some limitations in our study. As discussed earlier, eating disorders are rare in the general population. In our study sample, we identified ten persons with current disorder from 546 interviewed persons, and twenty with lifetime eating disorder who were currently in remission. Because of the small number of cases with current eating disorder in our general population sample, the confidence intervals of our estimates were relatively wide. This could have only been avoided by increasing the sample size considerably. The SCID interview also has its limitations. It is widely used for diagnosing psychiatric disorders, but the denial of the disease and non-disclosure by patients, common in eating disorders, may complicate identification of the disorders even in the SCID interview. On the other hand, we were able to utilize case notes and health care register data in our diagnostic assessment, making them exceptionally reliable for a general population study.

Altogether, the screening questionnaire was sent to 1863 persons and returned by 1316 (70.6%). This might have affected the generalizability of our results, if eating disorders were more common in non-responders. Our previous study showed that those who returned the MEAF questionnaire were younger, more often women, had more often graduated high school, and had less often than non-responders been treated for any mental health problem in a mental or general hospital according to the Finnish Hospital Discharge Register [16]. There were no other socioeconomic differences, nor differences in the frequency of psychiatric symptoms reported in the baseline interview [16]. However, although persons with hospital-treated disorders were less likely to return the questionnaire than others, we had access to their case notes and could ascertain their diagnoses. There was only one person with a lifetime history of eating disorder in this group, suggesting that the questionnaire non-response did not introduce any major bias in the study.

There was attrition in the MEAF interview as well: only 55.5% of those invited participated in the interview. However, attrition in the MEAF interview depended on age, sex, and education, but none of the scores in any of the screens we used for the mental health interview differed between interview participants and non-participants [16]. However, the item in SCOFF describing attitude related to appearance differed between questionnaire responders and non-responders. The difference was too small (9.1% vs. 13.4%) to introduce any major bias, but it may suggest that young people with a negative attitude toward their appearance are less likely to participate in a survey.

Even though the SCOFF did not prove to be an efficient tool in screening eating disorders from the general population, it could be a valid tool in ruling one out. When utilizing the threshold of one or more positive answers, all the eating disorder cases were detected giving us a sensitivity of 100%. This leads to conclusion that if a person has zero positive answers in the SCOFF questionnaire, it is very unlikely that she/he suffers from an eating disorder. The SCOFF could be a helpful tool in ruling out eating disorders in primary care and student health services.

## Conclusion

Detecting eating disorders among the general population has its own challenges. In such a population, the SCOFF could be used at most as an aid in ruling eating disorders out. Further studies are still needed to establish the performance and ideal target population of the SCOFF.

## Competing interests

The authors declare that they have no competing interests.

## Authors' contributions

SL drafted the manuscript. JMS conducted the statistical analyses. JMS, JP, TA-S and SIS did final diagnostic assessments. JMS, TA-S and JL obtained funding for the study. All authors contributed to the interpretation of data, participated in revising the manuscript for intellectual content, and approved the final version of the manuscript.

## Pre-publication history

The pre-publication history for this paper can be accessed here:



## References

[B1] Hoek HW (2006). Incidence, prevalence and mortality of anorexia nervosa and other eating disorders. Curr Opin Psychiatry.

[B2] Hsu LK (1996). Epidemiology of the eating disorders. Psychiatr Clin North Am.

[B3] Keski-Rahkonen A, Hoek HW, Susser ES, Linna MS, Sihvola E, Raevuori A, Bulik CM, Kaprio J, Rissanen A (2007). Epidemiology and course of anorexia nervosa in the community. Am J Psychiatry.

[B4] Hudson JI, Hiripi E, Pope HG, Kessler RC (2007). The prevalence and correlates of eating disorders in the National Comorbidity Survey Replication. Biol Psychiatry.

[B5] Steinhausen HC (2002). The outcome of anorexia nervosa in the 20th century. Am J Psychiatry.

[B6] Morgan JF, Reid F, Lacey JH (1999). The SCOFF questionnaire: assessment of a new screening tool for eating disorders. BMJ: British medical journal.

[B7] Garcia-Campayo J, Sanz-Carrillo C, Ibanez JA, Lou S, Solano V, Alda M (2005). Validation of the Spanish version of the SCOFF questionnaire for the screening of eating disorders in primary care. J Psychosom Res.

[B8] Garner DM, Garfinkel PE (1979). The Eating Attitudes Test: an index of the symptoms of anorexia nervosa. Psychol Med.

[B9] Garner DM, Olmstedt MP, Polivy J (1983). Development and validation of a multidimensional Eating Disorder Inventory for anorexia and bulimia. Int J Eat Disord.

[B10] Henderson M, Freeman CP (1987). A self-rating scale for bulimia. The 'BITE'. Br J Psychiatry.

[B11] Hautala L, Alin J, Liuksila P-R, Räihä H, Saarijärvi S (2006). SCOFF-syömishäiriöseulan reliabiliteetti ja rakennevaliditeetti murrosikäisten koululaisten seulonnassa. Duodecim.

[B12] Perry L, Morgan J, Reid F, Brunton J, O'Brien A, Luck A, Lacey H (2002). Screening for symptoms of eating disorders: reliability of the SCOFF screening tool with written compared to oral delivery. Int J Eat Disord.

[B13] Rodriguez Martin A, Novalbos Ruiz JP, Martinez Nieto JM, Escobar Jimenez L, Castro De Haro AL (2004). Epidemiological study of the influence of family and socioeconomic status in disorders of eating behaviour. Eur J Clin Nutr.

[B14] Rodriguez Martin A, Novalbos Ruiz JP, Martinez Nieto JM, Escobar Jimenez L, Castro de Haro AL (2005). Characteristics of eating disorders in a university hospital-based Spanish population. Eur J Clin Nutr.

[B15] Castaneda A, Suvisaari J, Marttunen M, Perälä J, Saarni S, Aalto-Setälä T, Aro H, Koskinen S, Lönnqvist J, Tuulio-Henriksson A Cognitive functioning in a population-based sample of young adults with a history of non-psychotic unipolar depressive disorders without psychiatric comorbidity. J Affect Disord.

[B16] Suvisaari J, Aalto-Setala T, Tuulio-Henriksson A, Harkanen T, Saarni SI, Perala J, Schreck M, Castaneda A, Hintikka J, Kestila L Mental disorders in young adulthood. Psychol Med.

[B17] Aromaa A, Koskinen S (2004). Health and Functional Capacity in Finland. Baseline Results of the Health 2000 Examination Survey. Publications of National Public Health Institute, B12, 2004.

[B18] Koskinen S, Kestilä L, Martelin T, Aromaa A (2005). Nuorten aikuisten terveys Terveys 2000-tutkimuksen perustulokset 18–29-vuotiaiden terveydestä ja siihen liittyvistä tekijöistä [The health of young adults Baseline results of the Health 2000 Study on the health of 18 to 29-year-olds and the factors associated with it].

[B19] Pirkola SP, Isometsa E, Suvisaari J, Aro H, Joukamaa M, Poikolainen K, Koskinen S, Aromaa A, Lonnqvist JK (2005). DSM-IV mood-, anxiety- and alcohol use disorders and their comorbidity in the Finnish general population – results from the Health 2000 Study. Social psychiatry and psychiatric epidemiology.

[B20] Goldberg DP, Gater R, Sartorius N, Ustun TB, Piccinelli M, Gureje O, Rutter C (1997). The validity of two versions of the GHQ in the WHO study of mental illness in general health care. Psychol Med.

[B21] Kessler RC, Barker PR, Colpe LJ, Epstein JF, Gfroerer JC, Hiripi E, Howes MJ, Normand SL, Manderscheid RW, Walters EE (2003). Screening for serious mental illness in the general population. Arch Gen Psychiatry.

[B22] Ewing JA (1984). Detecting alcoholism. The CAGE questionnaire. Jama.

[B23] WHO (1990). Composite International Diagnostic Interview (CIDI, Version 1.1).

[B24] WHO (1997). Composite International Diagnostic Interview (CIDI, Version 2.1).

[B25] Hirschfeld RM, Williams JB, Spitzer RL, Calabrese JR, Flynn L, Keck PE, Lewis L, McElroy SL, Post RM, Rapport DJ (2000). Development and validation of a screening instrument for bipolar spectrum disorder: the Mood Disorder Questionnaire. Am J Psychiatry.

[B26] First M, Anthony J, Tepper S, Dryman A (1997). Structured Clinical Interview for DSM-IV Axis I Disorders, Research Version, Nonpatient Edition (SCID-I/NP).

[B27] Luck AJ, Morgan JF, Reid F, O'Brien A, Brunton J, Price C, Perry L, Lacey JH (2002). The SCOFF questionnaire and clinical interview for eating disorders in general practice: comparative study. BMJ: British medical journal.

[B28] Hsiao JK, Bartko JJ, Potter WZ (1989). Diagnosing diagnoses. Receiver operating characteristic methods and psychiatry. Arch Gen Psychiatry.

[B29] (1999). SAS 8.02.

[B30] Eng J (2006). ROC analysis: web-based calculator for ROC curves. Baltimore: Johns Hopkins University [updated 2006 May 17; cited September 6, 2006]. http://www.jrocfit.org.

[B31] Cotton MA, Ball C, Robinson P (2003). Four simple questions can help screen for eating disorders. J Gen Intern Med.

[B32] Parker SC, Lyons J, Bonner J (2005). Eating disorders in graduate students: exploring the SCOFF questionnaire as a simple screening tool. J Am Coll Health.

[B33] Mond JM, Myers TC, Crosby RD, Hay PJ, Rodgers B, Morgan JF, Lacey JH, Mitchell JE (2008). Screening for eating disorders in primary care: EDE-Q versus SCOFF. Behav Res Ther.

[B34] Caamaño F (2002). Validation of SCOFF questionnaire among pre-teenagers. BMJ.

[B35] Rueda JGE, Diaz LA, Campo A, Barros JA, Avila GC, Orostegui LT, Osorio BC, Cadena Ldel P (2005). [Validation of the SCOFF questionnaire for screening of eating disorders in university women]. Biomedica.

[B36] Rueda JGE, Diaz Martinez LA, Ortiz Barajas DP, Pinzon Plata C, Rodriguez Martinez J, Cadena Afanador LP (2005). [Validation of the SCOFF questionnaire for screening the eating behaviour disorders of adolescents in school]. Aten Primaria.

[B37] Boyd JC (1997). Mathematical tools for demonstrating the clinical usefulness of biochemical markers. Scand J Clin Lab Invest Suppl.

[B38] Siervo M, Boschi V, Papa A, Bellini O, Falconi C (2005). Application of the SCOFF, Eating Attitude Test 26 (EAT 26) and Eating Inventory (TFEQ) Questionnaires in young women seeking diet-therapy. Eat Weight Disord.

[B39] Ogg EC, Millar HR, Pusztai EE, Thom AS (1997). General practice consultation patterns preceding diagnosis of eating disorders. Int J Eat Disord.

[B40] Lähteenmäki S, Saarni SE, Aalto-Setälä T, Perälä J, Saarni SI, Aro H, Lönnqvist J, Suvisaari J (2008). Comorbidity and prevalence of eating disorders in young adult population and a validation of SCOFF screen. Poster presentation Number NR1-035, Annual Meeting Of the American Psychiatric Association 2008 Washington DC.

[B41] Selzer R, Hamill C, Bowes G, Patton G (1996). The branched eating disorders test: validity in a nonclinical population. Int J Eat Disord.

[B42] Stice E, Telch CF, Rizvi SL (2000). Development and validation of the Eating Disorder Diagnostic Scale: a brief self-report measure of anorexia, bulimia, and binge-eating disorder. Psychological assessment.

[B43] Ghaderi A, Scott B (1999). Prevalence and psychological correlates of eating disorders among females aged 18–30 years in the general population. Acta Psychiatr Scand.

[B44] Ghaderi A, Scott B (2002). The Preliminary Reliability and Validity of the Survey for Eating Disorders (SEDs): A Self-Report Questionnaire for Diagnosing Eating Disorders. European Eating Disorders Review.

[B45] Mond JM, Hay PJ, Rodgers B, Owen C, Beumont PJ (2004). Validity of the Eating Disorder Examination Questionnaire (EDE-Q) in screening for eating disorders in community samples. Behav Res Ther.

[B46] Jacobi C, Abascal L, Taylor CB (2004). Screening for eating disorders and high-risk behavior: caution. Int J Eat Disord.

